# Artificial Intelligence for Classifying the Relationship between Impacted Third Molar and Mandibular Canal on Panoramic Radiographs

**DOI:** 10.3390/life13071441

**Published:** 2023-06-26

**Authors:** Antonio Lo Casto, Giacomo Spartivento, Viviana Benfante, Riccardo Di Raimondo, Muhammad Ali, Domenico Di Raimondo, Antonino Tuttolomondo, Alessandro Stefano, Anthony Yezzi, Albert Comelli

**Affiliations:** 1 Section of Radiological Sciences, Department of Biomedicine, Neuroscience and Advanced Diagnostics, University of Palermo, 90127 Palermo, Italy; antonio.locasto@unipa.it; 2 Ri.MED Foundation, Via Bandiera 11, 90133 Palermo, Italy; vbenfante@fondazionerimed.com (V.B.); amuhammad@fondazionerimed.com (M.A.); acomelli@fondazionerimed.com (A.C.); 3 Department of Health Promotion, Mother and Child Care, Internal Medicine and Medical Specialties, Molecular and Clinical Medicine, University of Palermo, 90127 Palermo, Italy; domenico.diraimondo@unipa.it (D.D.R.); bruno.tuttolomondo@unipa.it (A.T.); 4 Institute of Molecular Bioimaging and Physiology, National Research Council (IBFM-CNR), 90015 Cefalù, Italy; alessandro.stefano@ibfm.cnr.it; 5 Postgraduate Section of Periodontology, Faculty of Odontology, University Complutense, 28040 Madrid, Spain; info@rosariodiraimondo.it; 6 Postgraduate Section of Oral Surgery, Periodontology and Implant, University Sur Mississippi, Spain Istitutions, 28040 Madrid, Spain; 7 Department of Electrical and Computer Engineering, Georgia Institute of Technology, Atlanta, GA 30332, USA; anthony.yezzi@ece.gatech.edu

**Keywords:** contact relationship, convolutional neural network, inferior alveolar nerve, mandibular third molar, panoramic radiograph, ResNet-152, VGG-19

## Abstract

The purpose of this investigation was to evaluate the diagnostic performance of two convolutional neural networks (CNNs), namely ResNet-152 and VGG-19, in analyzing, on panoramic images, the rapport that exists between the lower third molar (MM3) and the mandibular canal (MC), and to compare this performance with that of an inexperienced observer (a sixth year dental student). Utilizing the k-fold cross-validation technique, 142 MM3 images, cropped from 83 panoramic images, were split into 80% as training and validation data and 20% as test data. They were subsequently labeled by an experienced radiologist as the gold standard. In order to compare the diagnostic capabilities of CNN algorithms and the inexperienced observer, the diagnostic accuracy, sensitivity, specificity, and positive predictive value (PPV) were determined. ResNet-152 achieved a mean sensitivity, specificity, PPV, and accuracy, of 84.09%, 94.11%, 92.11%, and 88.86%, respectively. VGG-19 achieved 71.82%, 93.33%, 92.26%, and 85.28% regarding the aforementioned characteristics. The dental student’s diagnostic performance was respectively 69.60%, 53.00%, 64.85%, and 62.53%. This work demonstrated the potential use of deep CNN architecture for the identification and evaluation of the contact between MM3 and MC in panoramic pictures. In addition, CNNs could be a useful tool to assist inexperienced observers in more accurately identifying contact relationships between MM3 and MC on panoramic images.

## 1. Introduction

The most frequent oral surgery operation is the extraction of the lower third molar [1], and it is well recognized that its surgical removal carries a possibility of damage to the inferior alveolar nerve (IAN) [2]. The common imaging approach for assessing impacted lower third molars is panoramic radiography (PR), which can offer information on the closeness of the tooth’s roots to the mandibular canal (MC) from a two-dimensional perspective. This knowledge is crucial since the most probable risk factor for IAN damage is the anatomical proximity between these two structures [3].

However, PR has several limitations, including anatomical noise, superimposition, and geometric distortion [4]. Cone beam computed tomography (CBCT) is suggested prior to the removal of the lower third molar when this one and the mandibular canal are overlaid on panoramic radiographs in order to minimize the risk of IAN injury [5], but due to a significant increase in economic costs and radiation exposure for the patient, this test is rarely utilized for regular evaluation [4]. In addition, for inexperienced observers, such as a dental student, evaluating the anatomical proximity of the third molar’s roots and mandibular canal on a panoramic radiograph can be difficult. To guarantee comprehensive assessment and failure to notice serious diseases and pathologies, requests for decision-support systems (DSS) in the field of maxillofacial imaging have increased in recent years [6,7], specifically convolutional neural network (CNN)-based deep learning (DL) systems [8]. CNNs are capable of a wide range of assignments, including semantic segmentation, object detection, and classification [9]. Starting with an image provided as input, CNN will provide the correct output of numerous classes learned. Several studies have been conducted on the possible applications of artificial intelligence in dentistry. For the diagnosis of dental caries [10], a total of 3000 periapical radiographs of different dental elements were divided into two groups (“caries” vs. “non-caries”) based on the radiographic report. Then the images were reevaluated by four dentists and provided as input to the CNN, which calculated the performance. In periodontology, 100 orthopantomographs were annotated by three experienced periodontists, who evaluated and marked as “periodontally compromised” or “healthy” each element in the radiograph. In order to evaluate images, the following metrics were calculated for the CNN: sensitivity, specificity, and F-measure [11]. In another study, Lee et al. [12] evaluated the diagnostic accuracy of a CNN in the diagnosis of periodontopathic dental elements from a dataset of 1740 periapical radiographs, divided into three groups according to disease severity, by three periodontists; in the oral pathology field, Yang et al. [13] compared the diagnostic performance of a CNN with that of a group of oral surgeons and a group of general dentists. They used 1603 OPTs divided into three groups (dentigerous cyst, ameloblastoma, keratocyst, and no lesion) based on the histopathological report of the lesion. The performance of the three assessments (CNN, surgeons, and general dentists) was calculated in terms of sensitivity, specificity, accuracy, and F1-score. Instead, in another study, the aim was to diagnose various lesions (dentigerous cysts, periapical cysts, ameloblastomas, and odontogenic keratocysts) on orthopantomograms. In this case, the initial dataset of 1282 OPTs was manually annotated and divided according to histopathological diagnosis into five groups. CNN was evaluated for both lesion detection and diagnosis [14]; instead, Ohashi et al. [6] investigated whether a CAD system applied to OPTs could improve the diagnostic performance of dentists with little experience in diagnosing maxillary odontogenic sinusitis; regarding temporomandibular joint disorders, Choi et al. [14] evaluated the ability of a neural network to detect changes in the articular heads of the TMJ on panoramic images. In this case, the CBCT radiology report was used as a gold standard for training the neural network used [14,15]. Of the previously listed studies, we can classify the different characteristics that distinguish them. In particular, we observe that the most commonly used type of image is panoramic images (n = 7), followed by periapical radiographs (n = 3). The number of images used for network training ranges from a maximum of 1740 [12] to a minimum of 98 [6]. The most commonly used image format is JPEG (n = 10), with a minimum image resolution of 299 × 299 pixels [10] and a maximum of 1976 × 976 pixels [16]. Among the neural networks used to perform these studies, namely VGG-19 [17] and ResNet-152 [18], we observe a variety of CNNs, notably YOLOv4 (n = 1), AlexNet (n = 1), GoogleNet (n = 2), VGG16 (n = 1), InceptionV3 (n = 1), ResNet101 (n = 1), YOLOv3 (n = 1), YOLOv2 (n = 1), and unspecified (n = 3). Regarding the choice of gold standard, we can observe how expert clinicians opinions (n = 4), radiological reports (n = 4), or histopathological reports (n = 2) were used to establish ground truth and create a label to be assigned to the images used in the study. As we can see from this brief review of the relevant literature, there has been no standard method for conducting this type of investigation until now.

In the above studies, the CNNs demonstrated high diagnostic performance, comparable to that of an experienced professional. Our study is in addition to a few others [5,19] that aimed to evaluate the diagnostic capabilities of a CNN in the specific task of classifying the relationship between MC and MM3 on panoramic radiographs.

In conclusion, this paper aims to understand whether a CNN may prove useful to bridge the experiential gap between a practitioner in training and a more experienced one. To investigate this, we have evaluated the diagnostic performance of two CNNs (namely, ResNet-152 and VGG-19) for the assessment of the anatomical connection between the MM3 and MC in panoramic radiographs, and we have compared the diagnostic capabilities of these two networks with those of a sixth year dental student with minimal experience in the aforementioned assessment.

## 2. Materials and Methods

The following study was articulated into three parts: in the first part, a clinician with twenty years of experience in dental radiology and a sixth year dental student independently studied and labeled a dataset of panoramic images of lower third molars, distinguishing the group of images presenting a contact between tooth roots and the mandibular canal from those in which no such contact was present. The classification performed by the experienced radiologist was chosen as the gold standard for the study.

In the second part, the same dataset of images was analyzed by two convolutional neural networks, which, after initial training, classified the images by assigning them to the “contact” or “non-contact” groups.

Finally, in the third part, the diagnostic performance of the CNNs was evaluated, comparing it with the gold standard and with the evaluation performed by the dental student.

Below, we explain the procedure in detail.

### 2.1. Patients

Eighty-three panoramic radiographs, including 142 third molars, were selected based on the following criteria: (1) the presence of not less than one lower third molar with fully developed dental roots; and (2) a well-depicted mandibular canal. Panoramic images were acquired with two devices: a Planmeca ProMax 3D (Planmeca Oy, Helsinki, Finland) operating at 60–90 kVp, 1–14 mA, and 9–37 s exposure time, and a Kavo OP3D Pro (Kavo Dental, Biberach, Germany) operating at 57–90 kVp, 3.2–16 mA, and 8.1–16.1 s exposure time.

To define the relationship between the MM3 and the MC, the existence of the following radiographic marks was assessed: (1) shadowing of the root, (2) disruption of the upper cortical line of the mandibular canal, and (3) deviation of the mandibular canal [20]. As shown in several studies in the literature [20,21,22,23,24,25,26], a contact between the mandibular third molar and the mandibular canal is shown by the presence of one or more of the aforementioned signs. Based on these standards, a radiologist with more than 20 years of expertise in dentomaxillofacial radiology and a sixth year dental student independently assessed the collection of images, labeled the images, and divided them into two groups: (1) no overlap or contact between the mandibular third molar and the canal (63 third molars, included in the non-contact group); and (2) contact or overlap (79 third molars, included in the contact group). According to the expert radiologist’s assessment, which was chosen as the gold standard for network training, 63 teeth (44.4%) were assigned to group 0 (no-contact) and 79 teeth (55.6%) to group 1 (contact).

### 2.2. Preparation of the Dataset

**Figure 1:**
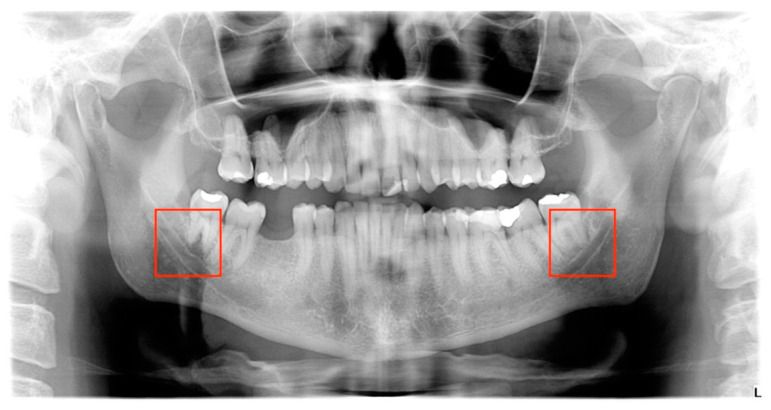
A panoramic radiograph with two red boxes designating the regions of interest (ROI). A location close to the canal and molar was chosen for the ROI’s center. The 2137 × 1024 pixel panoramic image was cropped to 224 × 224 pixels.

All panoramic radiographs, downloaded in DICOM3 format, were converted into NIfTI format. After the conversion, all the images were resized to a dimension of 2137 × 1024 pixels with the software 3DSlicer (Slicer, Brigham and Women’s Hospital, Harvard University, NIH). This dimension was chosen because it was the most prevalent in the image dataset. From the resized images, image areas with 224 × 224 square regions of interest were cut (Figure 1). The spot where the roots of the lower third molar and the mandibular canal were located most closely was designated the center of the region of interest (ROI).

Utilizing the k-fold cross-validation technique, 142 cropped images were divided into 80% training and validation images and 20% test images [27]. Specifically, the training-validation dataset, containing 80% of the images, was divided into five sub-datasets of equal numerosity, called folds, keeping in each the same proportion of cases belonging to “group 0” and “group 1” of the whole dataset. One of these datasets was used as a validation set to track the model’s performance during training. The other four were used as training sets. Then, the CNN was trained using the training datasets and monitored using the validation datasets. This technique was carried out five times, using a different fold for validation each time. Thus, the average performance obtained in the five training processes reveals the model’s accuracy. Finally, to calculate the final diagnostic performance, the test dataset was submitted to a CNN. This procedure was performed on both CNNs employed in the study, namely VGG-19 and ResNet-152.

### 2.3. Diagnostic Performance

The diagnostic accuracy of the two CNNs and the dental student was calculated using sensibility (TP/(TP+FN)), specificity (TN/(TN+FP)), Positive Predictive Value (TP/(TP+FP)), and accuracy ((TP+TN)/(TP+TN+FP+FN)).

TP (true positive), TN (true negative), FP (false positive), and FN (false negative).

### 2.4. Statistical Analysis

The one-way analysis of variance (ANOVA) and the post-hoc Tukey Honestly Significant Difference (HSD) test were used for statistical analyses [28]. ANOVA establishes whether there are statistically significant differences between the means of at least three independent datasets. This is achieved by comparing the variability within these datasets with the variability between the datasets. In this way, the averages between VGG-19, ResNet-152, and the dental student are compared to determine if one of these averages is statistically different from the others. A *p*-value less than 0.05 indicates that at least two means are significantly different from each other without indicating which dataset is significantly different from the others. Consequently, the HSD test is used to determine which dataset pairs are significantly different from each other.

## 3. Results

**Figure 2:**
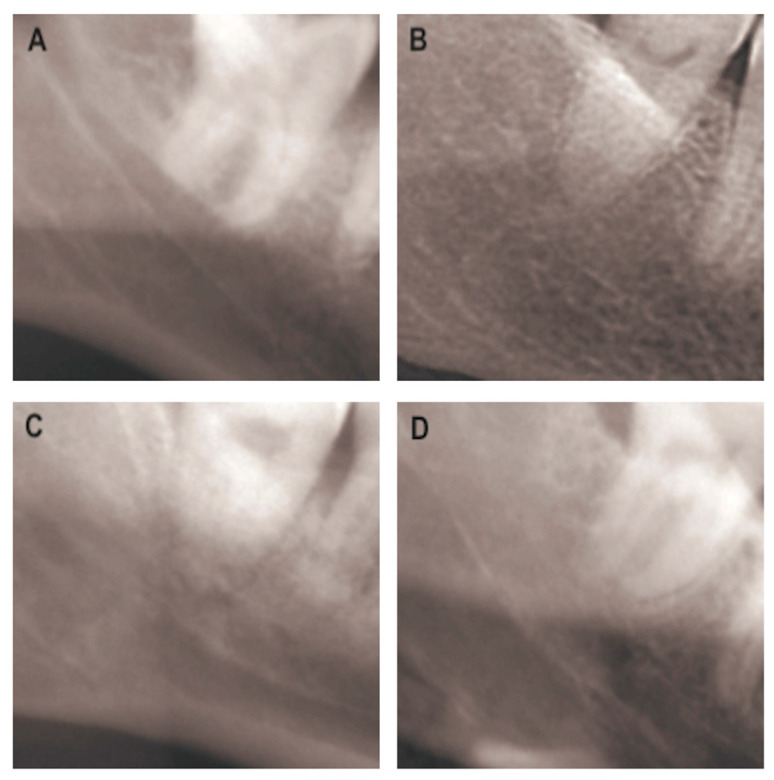
Example cases: (**A**) difficult case; in this case, the student performed an incorrect assessment, while both networks performed a correct assessment; (**B**) easy case; in this case, both the student and networks performed a correct assessment; (**C**) medium difficulty case; in this case, the student performed an incorrect assessment, while both networks performed a correct assessment; and (**D**) medium difficulty case; in this case, both the student and networks performed a correct assessment.

After training, validation, and testing of 142 images with the two CNNs used, the following results (Table 1) were obtained: for the VGG-19 network, 72.82% average sensitivity, 93.33% average specificity, 92.26% average positive predictive value, and 85.28% average accuracy; for the ResNet-152 network, 84.09% average sensitivity, 94.11% average specificity, 92.11% average positive predictive value, and 88.86% average accuracy. Therefore, VGG-19 and ResNet-152 showed comparable performance. Figure 2 shows some example cases. The sixth year dental student scored 69.60% sensitivity, 53.00% specificity, 64.85% positive predictive value, and 62.53% accuracy.

Lastly, a *p*-value lower than 0.05 (Table 2) indicated that the dentistry student and CNN algorithm results were significantly different. Specifically, the Tukey HSD test indicated which pairs were significantly different from each other, as shown in Table 3.

## 4. Discussion

The most common oral surgery procedure is the extraction of the lower third molar. This extraction, like any other surgery, may cause problems. The most serious complications after this operation are IAN damage. This injury can lead to neurosensory impairment of the innervated area, which negatively affects the patient’s quality of life [29]. To minimize the possibility of nerve damage, it is crucial to establish the lower molar’s relationship with the mandibular canal during the diagnosis stage. The common imaging approach for assessing an impacted lower third molar is panoramic radiography (PR), which can offer information on the closeness of the tooth’s roots to the mandibular canal (MC) from a two-dimensional perspective. Skilled dentists use the following marks to demonstrate the intimate connection between the lower third molar’s root and the mandibular canal: disruption of the upper cortical line of the mandibular canal, root shadowing, or mandibular canal deviation [21].

In this study, CNNs were used to detect such radiographic marks on panoramic radiographs. As shown in previous studies [5,12,19,30,31,32,33,34], the diagnostic capabilities of the CNNs utilized in our investigation were comparable to those of an expert in dentomaxillofacial radiology with more than 20 years’ expertise.

The dentistry student, however, got considerably lower results than both CNNs and the skilled radiologist. We can interpret these results by saying that, particularly for inexperienced observers, CNNs may be able to improve the diagnostic evaluation of the relationship between these two anatomical structures on panoramic images. Our study is in addition to a few others [5,19] that aim to assess the capabilities of a CNN for the specific task of classifying the connection between the mandibular canal and the lower third molar on panoramic radiographs. Specifically, in the study by Zhu et al. [19]., they compared the diagnostic capabilities of a convolutional neural network, called MM3-IANnet, based on YOLOv4, in classifying the relationship between the mandibular canal and the lower third molar. Initially, the network was trained from 503 panoramic images in JPEG format. CBCTs conducted on the same patient within three months of each other were considered the gold standard. The same panoramic images were initially analyzed by a CNN, then by five dentists with different experiences. Then the dentists were asked to reevaluate the images with the help of the network through a voting experiment. In this experiment, the dentist’s judgment had weight 1, and that of the network had weight 2.

Finally, the performance of the network, dentists, and network-aided dentists was compared. The results obtained in terms of average precision, precision, recall, and F1-score are, respectively, 83.02%, 88.71%, 91.67%, and 90.16% for MM3-IANnet; 76.45%, 89.95%, 83.00%, and 85.52% for dentists; and 88.06%, 93.88%, 92.00%, and 92.93% for the cooperative dentist-MM3-IANnet evaluation. In Fukuda’s study [5], however, a comparison of three different neural networks was performed: Alexnet, GoogLeNet, and VGG-16. These networks were initially trained on 600 panoramic images in JPEG format from a single database. In this case, unlike the previous study, the gold standard chosen was the qualitative evaluation of the same radiographic images by two radiologists. Once the results were obtained, the statistical performance of the three neural networks was calculated and compared. In addition to this, the time and storage space used by the three CNNs were calculated. The results obtained by the three neural networks used, calculated in terms of accuracy, sensitivity, and specificity, are, respectively, 0.90 ± 0.06, 0.88 ± 0.06, and 0.92 ± 0.05 for AlexNet; 0.92 ± 0.05, 0.88 ± 0.06, and 0.96 ± 0.04 for GoogLeNet; and 0.88 ± 0.06, 0.88 ± 0.06, and 0.88 ± 0.06 for VGG-16.

Based on the findings of our investigation, it is clear that the statistical performance of the CNNs utilized is completely equal to that of the studies by Zhu et al. [19] and Fukuda et al. [5], comparing sensitivity, specificity, positive predictive value, and accuracy. However, there are some differences in the study’s design. First, in the aforementioned studies, the format used by other authors Zhu et al. [19] and Fukuda et al. [5] for the input images is JPEG, whereas in the present study, we converted radiographic images obtained as DICOM files to the NIfTI format. This allowed us to reduce the loss of information from the original file compared to conversions to JPEG format. The network training performed with images in this format provided statistical performance comparable with previous studies. However, it was performed with a reduced sample of images: 915 (Zhu et al. [19]) and 600 (Fukuda et al. [5]) lower third molars, compared with the 142 molars included in our study. A further difference is the convolutional neural networks employed, which are different from those used in the studies by Zhu et al. [19] and Fukuda et al. [5]. In this context, the study published by Fukuda et al. [5] demonstrates how, when comparing the capabilities of three neural networks (VGG-16, GoogLeNet, and AlexNet) trained with the same dataset, the results provided are perfectly superimposable, with the only difference in terms of the processing time required, which varies according to the number of layers in the network. This was also confirmed in the present study, where the two convolutional networks used, ResNet-101 and VGG-19, provided comparable diagnostic performance (Table 3). As a result of a literature search of studies published between 2015 and 2022 in the PubMed database, using the keywords “CNN” and “Dentistry”, we decided to use VGG-19 [17], a deep convolutional neural network developed and trained by A. Zisserman and K. Simonyan of Oxford University at the ILSVRC-2014 (ImageNet Large Scale Visual Recognition Challenge 2014), as it was the most widely used in the studies included in the literature search. ResNet-152, an artificial neural network presented in 2015 by K. He, X. Zhang, and S. Ren [18], was also employed because it represented one of those with the largest number of layers. In the future, it would be useful to perform a comparative analysis including more of the existing CNNs to determine which among them obtains the best results in the analysis of radiographic images and whether this is related to particular features of the algorithm in question.

The study has other drawbacks in addition to the one mentioned. First, only two institutions provided panoramic images. To create more precise CNN models, images should be obtained from more institutions and from more PR devices. Second, an expert radiologist’s judgment rather than a three-dimensional (CT or CBCT) evaluation was used to identify the connection between the two structures. This would give the possibility of a more comprehensive approach to the problem of the relationship between tooth and canal; in particular, the evaluation of the position by CBCT would allow the distinction of different positions assumed by the roots with respect to the canal. In this case, a more detailed classification of the images could be obtained with an accurate anatomical position of the two structures. This is not just a dichotomous approach to distinguishing between “contact” and “noncontact”, as shown in the present study. Finally, to date, there are no other articles in the literature in which the performance of a CNN was compared with that of a trainee practitioner, i.e., a sixth year dental student, in evaluating the connection between the mandibular third molar and the mandibular canal. In our view, this type of comparison is critical to understanding how DSS can help the training of students and professionals. Zhu et al. [19] compared MM3-IANnet capabilities with those of five dentists with experience between 1 and 3 years. Next, dentists were asked to reevaluate the radiographs using CNN support. As a result, metrics were established to compare the deep learning network’s performance with dentists’ subjective evaluations and the collaborative dentist-MM3-IANnet approach. Instead, Fukuda et al. [5] compared exclusively the performance of the three CNNs they used to conduct the study (VGG-16, GoogLeNet, and AlexNet). In the present study, the capabilities of the neural networks employed were compared with those of a sixth year dental student, who is thus close to starting clinical practice. Indeed, we observed how image classification performed by both neural networks falls somewhere in between in terms of sensitivity, specificity, positive predictive value, and accuracy. This is between that performed by a radiologist with more than twenty years’ experience in dental and maxillofacial radiology and that of a sixth year dental student. From the results obtained, it is possible to state that convolutional neural networks can be integrated into a DSS for the classification of dental radiographic images, which generally require some experience by the dentist to be fully understood, thus succeeding in bridging the experiential gap between a trainee practitioner and one with more clinical experience.

## 5. Conclusions

The results obtained in our investigation have shown how the statistical performance of the neural networks used in radiological image classification is very close to that of an experienced radiologist in the field, as already observed by some studies in the literature. Moreover, since this performance is superior to that of a trainee practitioner, it is possible to affirm how the use of these DSS is useful for possibly bridging the latter’s experiential gap with a more experienced clinician. For example, future integration of artificial intelligence tools into a DICOM viewer application. In this way, a practitioner in training, in front of a similar diagnostic problem and in situations where a more experienced colleague is not available to confront him, can refer to the evaluation performed by the neural network to guide his decision-making, always keeping in mind that an expert clinician’s evaluation will never be substituted.

## Figures and Tables

**Table 1:** Comparison of statistical performance.

	Sensibility	Specificity	PPV ^1^	Accuracy
VGG-19				
Mean	72.82%	93.33%	92.26%	85.28%
±Std ^2^	20.81%	6.32%	8.05%	4.13%
±CI ^3^ (95%)	18.24%	5.54%	7.05%	3.62%
ResNet-152				
Mean	84.09%	94.11%	92.11%	88.86%
±Std	12.31%	7.21%	8.39%	7.38%
±CI (95%)	10.79%	6.32%	7.36%	6.47%
Student				
Mean	69.60%	53.00%	64.85%	62.53%
±Std	11.49%	9.59%	9.41%	9.40%
±CI (95%)	10.07%	8.41%	8.25%	8.24%

^1^ Positive Predictive Value; ^2^ Standard deviation, ^3^ Confidence interval.

**Table 2:** Tukey HSD (post-hoc test) was used as a multiple comparison method.

ANOVA	F-Value	F-Critical Value	*p*-Value
ResNet-152 vs. VGG-19 vs. Student	19.134	3.885	0.000185

**Table 3:** ANOVA on the DSC showed statistically significant differences between the results.

	Tukey HSD	Tukey HSD	Tukey HSD
Pair	Q statistic	*p*-value	Interference
ResNet-152 vs. VGG-19	1.0961	0.714887	*p* > 0.05
ResNet-152 vs. Student	8.0631	0.001005	*p* < 0.01
VGG-19 vs. Student	6.9669	0.001005	*p* < 0.01

## Data Availability

Data will be made available by the corresponding authors upon reasonable request.
